# Miscellany

**Published:** 1902-09

**Authors:** 


### Miscellany.

BACTERIA ON MONT BLANC.—In the *Comptes Rendus* of the Paris Academy of Sciences, M. Jean Binot prints an account of his researches in the observatory on the summit of Mont Blanc, where he has been conducting bacteriological investigations at the highest altitude yet explored. As was to be expected, the air on the summit, away from the observatory, contains scarcely any bacteria whatever, only from four to eleven being detected in as much as a thousand litres, while in somewhat similar volumes none whatever was found. As a rule, at lower altitudes the number of bacteria increased, as, for instance, at the Place de l'Aiguille fourteen, and at Montauvert forty-nine, were found in a thousand litres. Inside the observatory, in which M. Binot spent five days, from two hundred and sixty to five hundred and forty microbes were found in the same volume of air, these being probably introduced by M. Binot and his companions during their temporary invasion of the building.

The investigations were not, however, confined to the air on the top of the mountain, but included also bacterial examinations of freshly fallen snow, old snow, ice on the surface and below, glacier water, and mountain streams. Freshly fallen snow, even when sampled in large quantities, frequently contained no bacteria whatever, while in snow which had lain for some time usually only from one to two individuals were discoverable per cubic centimetre.

At the foot of the glaciers the surface snow contained rather more, the number varying from six to sixty-five per cubic centimetre at the Mer de Glace.

Glacier water is usually very pure, and, like the glacier ice from which it is derived, was found to contain a number of yeasts and some streptothrix; but while high up such water contained but from three to eight bacteria per cubic centimetre, a stream at the foot of the Glacier des Bossons contained ninety-five, while the water of the river Arve, at Chamonix, was found to have as many as seven thousand five hundred and fifty per cubic centimetre. Altogether, M. Benot examined one hundred and twenty-one samples of air, ice, snow, and water, and isolated no less than three hundred different varieties of microbes, one-third of which number he was able to identify as having been already studied and described, and the residue are being carefully investigated by him at the present time. Even the alluring and beautifully clear and crystalline spring water on the Montauvert road was condemned by being found to contain a dozen virulent colon bacilli in a cubic centimetre. Doubtless this pollution was due to the cattle on the mountain.—*Scientific American.*

---

The Action of Liquid Air upon Bacteria.—Myer refers to the numerous investigations regarding the effect of zero temperatures on bacteria. According to most authors, the effect is purely an inhibitive one. He refers to the experiments of A. C. White and Macfadyen with liquid air.

White claimed that pure cultures of bacteria were not killed by exposure to the action of liquid air. Macfadyen found that liquid air acting on bacteria even as long as a week failed to kill them. Myer tried the effect of liquid air on anthrax spores, and on the staphylococcus pyogenes aureus. He exposed the organisms to the air in the form of a spray, or else put liquid air right on the culture, or put the test-tubes in the liquid air. The time of exposure varied from five seconds to fifteen minutes. In no instance did he find death of the bacteria; in fact, not even a loss of any of their properties resulted. He proposes to publish experiments later bearing on the action of liquid air on bacteria in inflamed tissue.—*Albany Medical Annals.*

Local Anæsthesia by High-Frequency Electrical Currents.—A method of replacing the ordinary anæsthetics used in dental surgery by the action of the high-frequency currents has been brought out by Messrs. Regnier and Didsbury, of Paris. M. d'Arsonval has already shown that high-tension and high-frequency currents have a local anæsthetic effect, and the experimenters wished to see whether this could not be used to advantage for dental operations, and so do away with the inhalations of gas, which are not without danger to the patient. In case of extraction they found it to work quite successfully. A d'Arsonval-Gaiffe apparatus was used, having a coil which gave a 1.2-inch spark with a rotary interrupter and an oil condenser. The apparatus is connected to an Oudin resonator, one of whose terminals is joined by a flexible cord to an electrode fixed under the jaw. The electrode is moulded in plastic material and covered inside by metallic powder and a layer of tin-foil.

Under these conditions the current gave the patient no sensation other than a slight heating in the region covered by the electrodes. It was found that a tooth with one root was made completely insensible by the application of a current of one hundred and fifty milliampères for from three to five minutes, while the larger teeth needed from two hundred to two hundred and fifty milliampères for from six to eight minutes. As to the use of the method for more prolonged operations, the experiments are not as yet conclusive, although they are favorable on the whole.—*Scientific American.*

---

An Original Method of adapting Backings to Facings.—At the seventeenth annual meeting of the Minnesota State Dental Association, held at Minneapolis, in September, 1900, Dr. A. E. Peck demonstrated an original method of adapting backings to facings. An ordinary rubber block is used, with holes for the reception of the pins. The backing is placed on the tooth, which is then placed on the rubber block, the pins being inserted in the holes. Another rubber block is placed on the porcelain and struck with a mallet until the desired adaptation is obtained. There is no danger of breaking the tooth by the force of the blow.—*Dental Review.*

Diagnosis of Presence of Pus by Heat.—Lewin, of Berlin, claims that by the use of the local application of heat we can make a diagnosis as to whether an acute inflammatory process has gone on to suppuration,—as, for example, in case of appendicitis. He asserts that, if pus has not yet formed, the application of heat will be a comfort to the patient; whereas, on the other hand, if pus be present, it will so increase and exacerbate the pain that a diagnosis of the presence of this material can be made with assurance. He states as an example that, in cases of swelling of the knee associated with rheumatism or otherwise, we not infrequently are able to give great relief if the knee is put at rest with a fixation point and heat is actively employed. If by chance pus be present, the pain is augmented and becomes intolerable. Lewin states that he has employed heat for this purpose in a sufficient number of cases to make him feel confident that he cannot be mistaken in regard to this point, and cites ten cases of appendicitis in which the heat was applied for two hours by means of hot compresses and without the use of internal pain-relievers. Eight of these received this treatment with a good deal of relief, but the remaining two showed marked increase of pain.

Of the eight cases all went on to cure in the space of from five days to three weeks, while, on the contrary, the two which suffered an increase of pain after the application of heat required the administration of opium for the relief of pain, and both died.

Similar results were obtained by Sphor, of Frankfort-on-the-Main.

If further investigation show that this method of diagnosis is at all accurate, it is so simple in its application that it cannot fail to prove of value.—*Therapeutic Gazette.*

---

Pernicious Anæmia caused by Oral Sepsis.—In reviewing a little brochure written by William Hunter, M.D., F.R.C.P., entitled "Oral Sepsis as the cause of Septic Gastritis," the *Therapeutic Gazette* makes the following comment:

"This is practically a reprint of an article which appeared in the *London Practitioner* of December, 1900, and is designed to spread the views held by Dr. Hunter as indicated in the title. He believes, as some of our readers may perhaps know, that even certain cases of pernicious anæmia have their origin in oral sepsis, and it would seem exceedingly likely that filthiness about the mouth readily tends to produce general infection.

"While we are not able to agree with Dr. Hunter in many of the views which he advances, we think he has done a service in drawing attention to this important matter."

